# Clinical applications of intra-cardiac four-dimensional flow cardiovascular magnetic resonance: A systematic review

**DOI:** 10.1016/j.ijcard.2017.07.023

**Published:** 2017-12-15

**Authors:** Saul Crandon, Mohammed S.M. Elbaz, Jos J.M. Westenberg, Rob J. van der Geest, Sven Plein, Pankaj Garg

**Affiliations:** a Division of Biomedical Imaging, Leeds Institute of Cardiovascular and Metabolic Medicine (LICAMM), University of Leeds, Leeds, United Kingdom; b Leiden University Medical Centre, Leiden, The Netherlands

**Keywords:** Intra-cardiac, Systematic review, Four-dimensional, 4D flow CMR, 4D flow MRI, Cardiovascular magnetic resonance

## Abstract

**Background:**

Four-dimensional flow cardiovascular magnetic resonance (4D flow CMR) is an emerging non-invasive imaging technology used to visualise and quantify intra-cardiac blood flow. The aim of this systematic review is to assess the literature on the current clinical applications of intra-cardiac 4D flow CMR.

**Methods:**

A systematic review was conducted to evaluate the literature on the intra-cardiac clinical applications of 4D flow CMR. Structured searches were carried out on Medline, EMBASE and the Cochrane Library in October 2016. A modified Critical Skills Appraisal Programme (CASP) tool was used to objectively assess and score the included studies. Studies were categorised as ‘highly clinically applicable’ for scores of 67–100%, ‘potentially clinically applicable’ for 34–66% and ‘less clinically applicable’ for 0–33%.

**Results:**

Of the 1608 articles screened, 44 studies met eligibility for systematic review. The included literature consisted of 22 (50%) mechanistic studies, 18 (40.9%) pilot studies and 4 (9.1%) diagnostic studies. Based on the modified CASP tool, 27 (62%) studies were ‘highly clinically applicable’, 9 (20%) were ‘potentially clinically applicable’ and 8 (18%) were ‘less clinically applicable’.

**Conclusions:**

There are many proposed methods for using 4D flow CMR to quantify intra-cardiac flow. The evidence base is mainly mechanistic, featuring single-centred designs. Larger, multi-centre studies are required to validate the proposed techniques and investigate the clinical advantages that 4D flow CMR offers over standard practices.

PROSPERO = CRD42016051438.

## 1 Introduction

Cardiovascular disease is the leading cause of mortality worldwide, with an estimated 17.7 million deaths in 2015 alone [1]. Blood flow is a vital parameter in the assessment of cardiovascular disease and hence, requires precise measurement.

Non-invasive imaging techniques, such as echocardiography and two-dimensional cine phase contrast magnetic resonance imaging (2D PC MRI), are standard components of a thorough cardiovascular investigation. Despite this, current quantification methods cannot fully assess the complex, three-dimensional and multi-directional nature of intra-cardiac blood flow.

Four-dimensional flow cardiovascular magnetic resonance (4D flow CMR) is a technique that enables comprehensive haemodynamic flow assessments. In recent years, 4D flow acquisition and post-processing techniques have greatly advanced, accompanied by the development of several new quantification methods. The latest 4D flow consensus document supplies a detailed overview of current 4D flow uses, both cardiac and non-cardiac [2]. This has been supplemented by several other review articles [3], [4], [5]. However, existing articles tend to encompass a large breadth of applications. The most recent 4D flow reviews addressing cardiac applications specifically, are from 2011, featuring non-systematic methodologies [6], [7]. With the rapid advancement of 4D flow, a comprehensive and archival update is warranted. This is especially relevant for the ageing population, given that adult cardiac disease, such as heart failure and valvular heart disease, presents a major and increasing health burden [8], [9]. Therefore, the aim of this systematic review is to methodically summarise new mechanistic intra-cardiac findings, along with information regarding the potential clinical applications of these 4D flow CMR techniques.

## 2 Methods

This systematic review was registered in the international database of prospectively registered systematic reviews (PROSPERO, registration number = CRD42016051438). The review protocol can be accessed online via the PROSPERO website [10]. The Preferred Reporting Items for Systematic Reviews and Meta-Analysis (PRISMA) checklist was adhered to when structuring this article [11].

### 2.1 Search strategy

A comprehensive search was undertaken in October 2016 on the following electronic databases: Medline, EMBASE and the Cochrane Library. The search was limited to ‘Humans’ and ‘English Language’ only. There was no limitation on time periods. To minimise bias, OpenGrey and Copac databases were also searched to identify any grey literature. In addition, clinicaltrials.gov was searched to find any ongoing studies. The supplemental file provides an in-depth description of the search strategy. Citation tracking and manual reference searching was carried out through the OvidSP databases.

### 2.2 Article screening

**Fig. 1:**
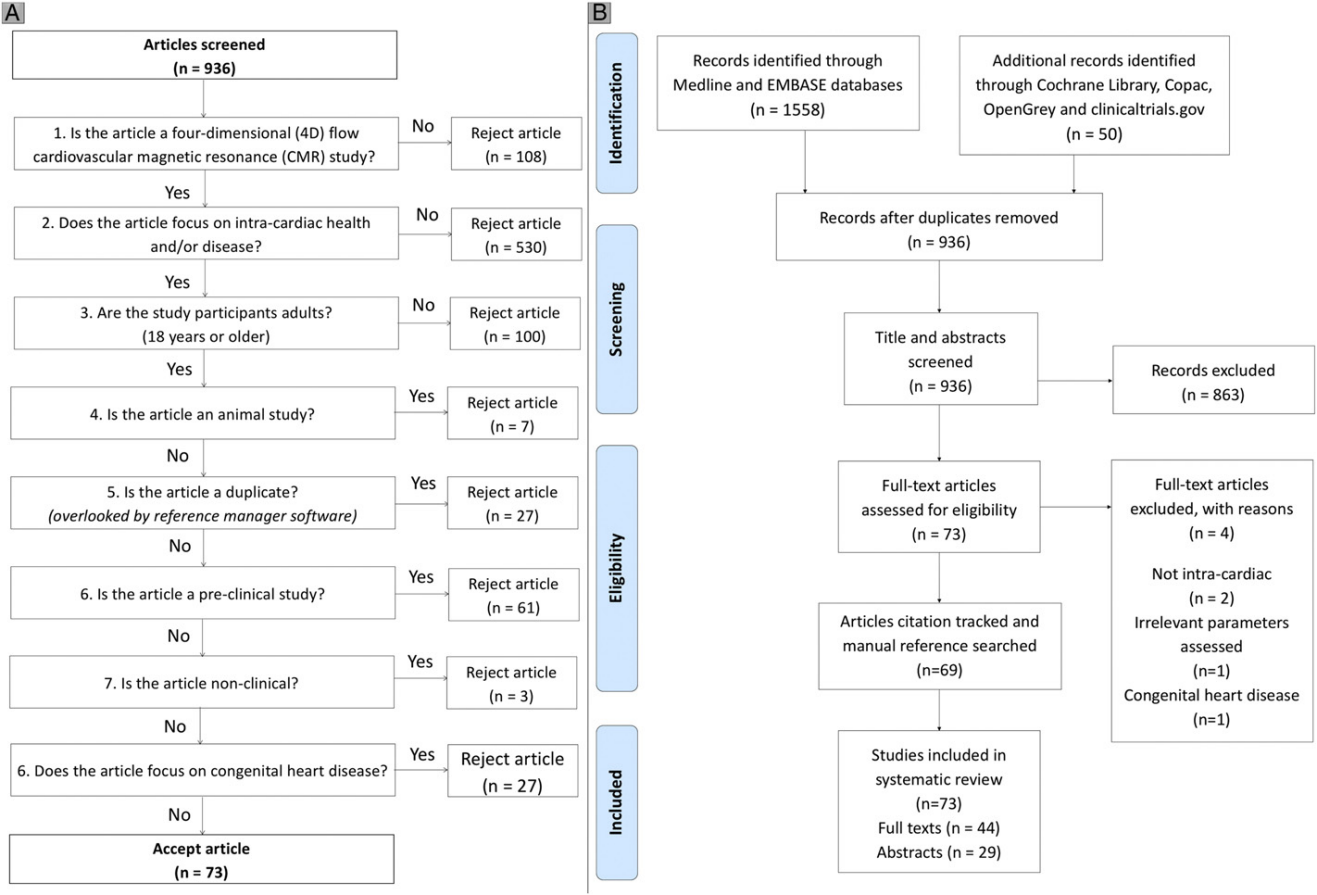
Overview of study selection process. Panel A = Article screening algorithm. Two reviewers independently screened the titles and abstracts of 936 studies using this system. The number of studies excluded at each stage is shown. Panel B = Flow diagram used for identifying the included studies. The full-texts of the 73 studies that were identified from the screening process were assessed. Of these, 4 (5.5%) were excluded as being irrelevant to the systematic review. The remaining 69 studies underwent citation tracking through the OvidSP databases, as well as manual reference searching. This process identified a further 4 relevant studies for inclusion. Of the final 73 studies included, 44 (60.3%) were full studies, whereas 29 (39.7%) were abstracts only. Flow diagram adapted from Moher et al. [11]. Preferred reporting items for systematic reviews and meta-analyses: The PRISMA Statement. PLOS Medicine 2009. 6 (7):e1000097. The PRISMA Statement is distributed under the terms of the Creative Commons Attribution License, which permits unrestricted use, distribution, and reproduction in any medium.

Once duplicates were removed, the titles and abstracts of the search results were assessed using a screening algorithm, shown in Fig. 1, Panel A. This was performed by two independent reviewers (S·C and P.G) and cross-checked against a third independent reviewer (M.E). Cases of disagreement were discussed between the reviewers to reach a suitable conclusion. The studies that adhered to this screening had their full texts evaluated.

### 2.3 Quality assessment

The quality of the included studies was assessed by P.G using a modified Critical Appraisal Skills Programme (CASP) tool, provided in the supplemental file. Answers of ‘yes’ scored 1 point, whereas answers of ‘no’ or ‘can't tell’ scored 0 points. Total scores were converted to percentages and studies were allocated to one of three categories; ‘highly clinical applicable’ for a score of 67–100%, ‘potentially clinically applicable’ for 34–66% and ‘less clinically applicable’ for 0–33%. Study quality was used as an estimate of clinical applicability for the purposes of this review.

### 2.4 Quantitative assessment

A meta-analysis was deemed inappropriate for this systematic review as much of the research is exploratory, with considerable heterogeneity in the included studies. As a result, a narrative review is provided.

## 3 Results

The search yielded a total of 1608 studies. These consisted of 539 (33.5%) and 1019 (63.4%) studies sourced from electronic databases Medline and EMBASE respectively, 18 (1.1%) from the Cochrane Library database, 20 (1.2%) from grey literature searches (Copac and OpenGrey databases) and 12 (0.7%) from clinicaltrials.gov.

Of the 1608 studies identified, 672 (41.8%) were removed as duplicates. After screening the title and abstracts of the remaining studies, a further 863 (53.7% of original 1608) were removed as irrelevant. Assessing the full texts of the remaining 73 studies resulted in 4 further exclusions. Citation tracking resulted in no new relevant studies whereas manual reference searching identified 4 relevant records. From the final 73 studies, 29 (39.7%) were abstracts and are presented in the supplemental file. The remaining 44 (60.3%) records were full-text studies. The PRISMA algorithm for study inclusion is presented in Fig. 1, Panel B.

### 3.1 Included studies

**Fig. 2:**
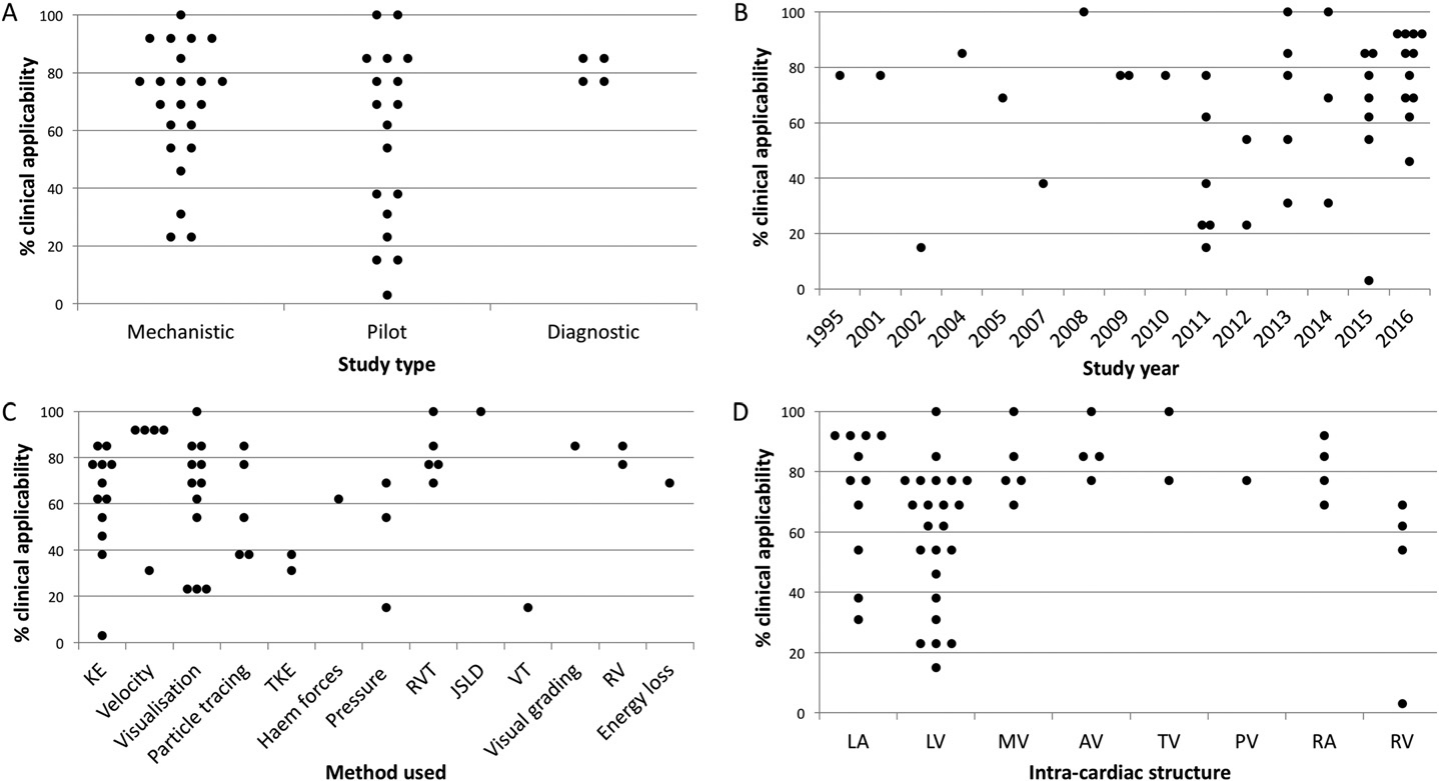
Graphical representations of the percentage of clinical applicability against various study design factors, Panel A = against study type, Panel B = against study year, Panel C = against the 4D flow methods used and Panel D = against intra-cardiac structure. KE = kinetic energy, TKE = turbulent kinetic energy, Haem forces = haemodynamic forces, RVT = retrospective valve tracking, JSLD = jet shear layer detection method, VT = volume tracking, RV = regurgitant volume, LA = left atrium, LV = left ventricle, MV = mitral valve, AV = aortic valve, TV = tricuspid valve, PV = pulmonary valve, RA = right atrium, RV = right ventricle.

In total, 44 studies were included in this systematic review. Of these, 22 (50%) were mechanistic studies, 18 (40.9%) were pilot studies and 4 (9.1%) were diagnostic studies. The modified CASP tool found that 27 (62%) studies were ‘highly clinically applicable’, 9 (20%) were ‘potentially clinically applicable’ and 8 (18%) were ‘less clinically applicable’. Graphs of the percentage of clinical applicability against various study design factors are depicted in Fig. 2, A to D. The studies were divided according to intra-cardiac structure, 34 of which were relevant to the left heart, 12 addressing the valves of the heart, and 8 for the right heart. Some studies were relevant to more than one category. All 44 included studies were single-centred. A summary of the included studies' characteristics for the left heart, heart valves and right heart can be found in Table 1. The breakdown of scoring for each study is provided in the supplemental file.

**Table 1:** Summary of included studies.

Author(s), year	Study type	Cohort	Methods	Applicability
Left atrium				
Lee et al., 2016 [12]	Mechanistic	30 HV, 40 AF	Velocity profile	Highly
Markl et al., 2016 [13]*	Retrospective mechanistic	8 HV, 62 AF	Velocity profile	Highly
Markl et al., 2016 [14]	Mechanistic	30 HV, 81 AF	Velocity profile and its maps	Highly
Markl et al., 2016 [15]	Retrospective mechanistic	15 HV, 60 AF	Velocity profile	Highly
Steding-Ehrenborg et al., 2015 [16]*	Retrospective mechanistic	14 HV, 14 athletes	KE	Highly
Suwa et al., 2015 [17]	Prospective mechanistic	9 HV, 15 No OHD, 17 OHD	Pathline visualisation of vortex	Highly
Arvidsson et al., 2013 [18]*	Mechanistic	15 HV	KE	Highly
Fluckiger et al., 2013 [19]	Pilot	19 HV, 10 AF	Flow velocity distribution	Less
Foll et al., 2013 [20]*	Mechanistic	24 HV	Vortex and flow visualisation	Potentially
Dyverfeldt et al., 2011 [21]	Pilot	2 HV, 5 MR	Particle tracing visualisation and turbulent KE	Potentially
Fyrenius et al., 2001 [22]	Mechanistic	11 HV	Particle tracing visualisation	Highly

Left ventricle				
Eriksson et al., 2016 [23]	Mechanistic	10 HV, 10 DCM	Global haemodynamic forces	Potentially
Suwa et al., 2016 [24]	Mechanistic	22 pEF, 14 rEF	Pathline visualisation of vortex	Highly
Svalbring et al., 2016 [25]	Mechanistic	10 HV, 26 IHD	KE	Potentially
Van Ooij et al., 2016 [26]	Mechanistic	10 HV, 35 HCM	Pressure gradients and viscous energy loss	Highly
Wong et al., 2016 [27]	Prospective mechanistic	35 HV, 10 rEF	KE	Highly
Al-Wakeel et al., 2015 [28]	Pilot	7 HV, 10 MR	KE	Potentially
Eriksson et al., 2015 [29]	Mechanistic	12 HV	Pressure gradients	Potentially
Kanski et al., 2015 [30]	Prospective mechanistic	12 HV, 29 HF	KE	Highly
Kanski et al., 2015 [31]	Pilot	8 HV, 20 HF	Particle tracing visualisation and kinetic energy	Highly
Steding-Ehrenborg et al., 2015 [16]*	Retrospective mechanistic	14 HV, 14 athletes	KE	Highly
Zajac et al., 2014 [32]	Mechanistic	11 HV, 9 DD	Turbulent KE	Less
Elbaz et al., 2014 [33]	Pilot	24 HV	Vortex visualisation	Highly
Eriksson et al., 2013 [34]	Prospective mechanistic	10 HV, 10 DCM	Pathline visualisation and KE	Highly
Foll et al., 2013 [20]*	Mechanistic	24 HV	Particle tracing and velocity vector visualisation	Potentially
Carlsson et al., 2012 [35]*	Pilot	9 HV	KE	Potentially
Toger et al., 2012 [36]	Mechanistic	9 HV, 4 DCM	Vortex visualisation	Less
Brandts et al., 2011 [37]	Prospective diagnostic	47 HF	Diastolic function using retrospective valve tracking	Highly
Eriksson et al., 2011 [38]	Mechanistic	12 HV, 1 DCM	Pathline visualisation and KE	Less
Kumar et al., 2011 [39]	Prospective pilot	15 DD	Flow visualisation	Less
Toger et al., 2011 [40]	Pilot	8 HV, 1 ALVA	Volume tracking	Less
Eriksson et al., 2010 [41]	Pilot	6 HV, 3 DCM	Pathline visualisation	Highly
Bolger et al., 2007 [42]	Pilot	17 HV, 1 DCM	Particle tracing and KE	Potentially
Ebbers et al., 2002 [43]	Pilot	1 HV	Pressure gradients	Less
Kim et al., 1995 [44]	Mechanistic	26 HV	Flow visualisation	Highly

Mitral valve				
Marsan et al., 2009 [45]	Diagnostic	64 MR	Regurgitant volume, 4D flow as reference method	Highly
Roes et al., 2009 [46]*	Pilot	22 HV, 29 IC	Valve flow quantification using retrospective valve tracking	Highly
Westenberg et al., 2008 [47]*	Retrospective pilot	10 HV, 20 MR/TR	Valve flow quantification using retrospective valve tracking	Highly
Westenberg et al., 2005 [48]	Pilot	10 HV, 10 MR	Valve flow quantification using retrospective valve tracking	Highly
Westenberg et al., 2004 [49]	Pilot	10 HV	Valve flow quantification using retrospective valve tracking	Highly

Aortic valve				
Chelu et al., 2016 [50]	Prospective diagnostic	54 AR	Visual grading of AR	Highly
Garcia et al., 2014 [51]	Retrospective pilot	10 HV, 40 AS	Jet shear layer detection method	Highly
Ewe et al., 2013 [52]	Retrospective diagnostic	32 AR	Regurgitant Volume with 4D flow as reference method	Highly
Roes et al., 2009 [46]*	Pilot	22 HV, 29 IC	Valve flow quantification using retrospective valve tracking	Highly

Tricuspid valve				
Roes et al., 2009 [46]*	Pilot	22 HV, 29 IC	Valve flow quantification using retrospective valve tracking	Highly
Westenberg et al., 2008 [47]*	Retrospective pilot	10 HV, 20 MR/TR	Valve flow quantification using retrospective valve tracking	Highly

Pulmonary valve				
Roes et al., 2009 [46]*	Pilot	22 HV, 29 IC	Valve flow quantification using retrospective valve tracking	Highly

Right atrium				
Callaghan et al., 2016 [53]	Prospective pilot	12 HV	Particle tracing and kinetic energy	Highly
Markl et al., 2016 [13]*	Retrospective mechanistic	8 HV, 62 AF	Velocity profile	Highly
Steding-Ehrenborg et al., 2015 [16]*	Retrospective mechanistic	14 HV, 14 athletes	KE	Highly
Arvidsson et al., 2013 [18]*	Mechanistic	15 HV	KE	Highly

Right ventricle				
Han et al., 2015 [54]	Pilot	9 HV, 10 PAH	KE	Less
Steding-Ehrenborg et al., 2015 [16]*	Retrospective mechanistic	14 HV, 14 athletes	KE	Highly
Carlsson et al., 2012 [35]*	Pilot	9 HV	KE	Potentially
Fredriksson et al., 2011 [55]	Mechanistic	10 HV	Pathline visualisation and KE	Potentially

Summary of the 44 included studies. Of these, 35 address the left heart, 12 for the heart valves and 8 for the right heart. Clinical applicability has been divided into the following groups based on the score achieved using the modified CASP tool: ‘highly’ for 67–100%, ‘potentially’ for 34–67% and ‘less’ for 0–33%. Studies that are relevant to more than one intra-cardiac structure are denoted by an asterisk (*). HV = healthy volunteers, AF = atrial fibrillation, OHD = organic heart disease, MR = mitral regurgitation, DCM = dilated cardiomyopathy, pEF = preserved ejection fraction, rEF = reduced ejection fraction, IHD = ischaemic heart disease, HCM = hypertrophic cardiomyopathy, HF = heart failure, DD = diastolic dysfunction, ALVA = apical left ventricular aneurysm. IC = ischaemic cardiomyopathy, TR = tricuspid regurgitation, AR = aortic regurgitation, AS = aortic stenosis. PAH = pulmonary arterial hypertension.

## 4 Discussion

To the authors' knowledge, this is the first systematic review of intra-cardiac 4D flow CMR. This systematic review incorporated 44 (2.7%) full papers and 29 (1.8%) abstracts from the 1608 records identified.

### 4.1 Left heart

#### 4.1.1 Left atrium

A 2001 study was one of the first to define normal left atrial (LA) flow patterns using particle trace visualisation [22]. These findings were supported by a 2015 study from Suwa et al. [17], who proposed that the presence of vortices in the LA may have a role in minimising blood stasis and thus, preventing thrombus formation. This concept has since been investigated widely by Markl et al. [13], [14], [15]. In a study investigating velocity profiles, patients with atrial fibrillation (AF) had 11–19% higher blood stasis compared to controls [13]. Taken further, velocity maps are reliable tools for detecting both regional and global flow patterns in the LA of AF patients [14]. Additionally, they found stasis was significantly higher (*P* < 0.001) at the wall of the LA, compared to the LA centre, a finding which complements preliminary work by Fyrenius et al. [22]. Recommendations by a third study [15] as well as Lee et al. [12], suggest the need for longitudinal studies to assess whether intra-atrial flow dynamics, as derived by 4D flow CMR, offer better predictions for thromboembolic events than the current, epidemiologically based, CHA_2_DS_2_-VASc score [19].

Dyverfeldt et al. assessed turbulent kinetic energy (TKE), in mitral regurgitation (MR) patients [21]. TKE encompasses the energy eventually lost as heat in turbulent flow. Mean LA TKE correlated with the severity of MR (r^2^ = 0.983, *P* < 0.001), however this study was limited to only 5 patients.

In health, younger individuals showed higher LA velocity inside the vortex than older individuals (*P* = 0.012) [20], suggesting that changes in intra-LA haemodynamics are part of normal ageing physiology. Arvidsson et al. found a weak correlation between early diastolic KE of the LA and left ventricular (LV) mass (r^2^ = 0.28, *P* < 0.05) [18]. They proposed that the elastic recoil of the LV causes a rise in LA KE in early diastole and so, diastolic suction is likely to be responsible for LV filling.

#### 4.1.2 Left ventricle

The LV is the most extensively studied structure within the intra-cardiac 4D flow literature. This began with Kim et al. identifying the presence of LV vortices as well as its close relationship with anterior mitral leaflet motion [44]. This validated the speculations produced by in vitro flow visualisation models at the time.

In recent years, various studies have used 4D flow CMR to further understand normal LV blood flow [20], [29], [33], [35], [43]. Foll et al. described the effects of multiple demographic variables on LV haemodynamics [20]. It was found that increasing age has an inverse relationship with the number of both LV vortices [(correlation coefficient (CC) = − 0.51, *P* = 0.01] and diastolic vortices (CC = − 0.49, *P* = 0.03). In addition, older subjects had reduced size and velocity within their basal LV vortices, whilst women had smaller basal LV vortices than men. Supplementing this, Wong et al. found older individuals have a lower peak diastolic KE compared with both children (*P* = 0.0001) and young adults (*P* = 0.025), suggesting a progressive decline with advancing age [27]. These studies highlight the wide variety of normal LV blood patterns.

Al-Wakeel et al. used KE for the assessment of surgical outcomes in MR patients [28], finding significant decreases in mean KE, systolic and early-diastolic KE peaks after mitral valve (MV) repair (*P* = 0.01, 0.02 and 0.01 respectively). A larger study found that heart failure patients had lower average normalised systolic KE when compared with healthy volunteers (6.3 ± 2.2 mJ/ml vs 8.0 ± 2.1 mJ/ml, *P* = 0.025) [30]. Carlsson et al. quantified the KE for the left and right ventricle, noting higher early diastolic KE in the LV (6.0 ± 0.6 mJ vs 3.6 ± 0.4 mJ, *P* = 0.004) [35]. This indicates that LV filling is more dependent on suction mechanisms. Unlike previous studies, these values weren't normalised for LV volumes.

KE has been proposed as a subclinical marker of LV dysfunction in dilated cardiomyopathy (DCM) patients, following observations that alterations in diastolic haemodynamics can be detected despite clinical compensation [34]. Moreover, ischaemic heart disease patients display reductions in ‘Direct flow’ and KE at end-diastole as LV volumes increase, compared to controls [25]. Changes in ‘these parameters may provide more sensitive clinical classifications of LV dysfunction over standard volumetric assessments.

Many studies have compared normal ventricular function with that of DCM patients [23], [34], [36], [38], [41], [42]. One study described the reduced efficiency of a DCM heart, preserving only 5% of the mitral inflow KE compared with 16% for healthy volunteers [42]. However, these are anecdotal findings, given this study featured only one patient. Eriksson et al. demonstrated that LV haemodynamic filling forces of DCM hearts are non-uniform [23] and the volume of LV inflow that is directly ejected, is decreased [34], [38]. The group also mapped out normal LV pressure differences [29] as well as validating semi-automatic quantification analysis as an accurate and reproducible method of assessing LV inflow and outflow volumes [41].

Colour vector analysis was proposed as a novel method of assessing LV diastolic flow in a study of 15 patients with diastolic dysfunction, by observing the termination of organised high velocity flow at the mid-LV [39]. Vortex size and vortex core locations were analysed between heart failure patients, in both preserved and reduced ejection fraction [24]. This study suggests diastolic vortex formation may be crucial for the LV ejection, as well as filling. Characteristic haemodynamic changes need to be established before this can be introduced clinically.

Volume tracking has been offered as an additional way to quantify blood flow [40]. It was tested against particle tracing, a common 4D flow CMR visualisation method. Volume tracking showed an average of 90.5% agreement with particle tracing in mid-diastole as well as strong inter-observer agreement (κ = 0.91). Despite this, the clinical applicability of this method is uncertain given its potential to accrue velocity errors and the unknown influence of factors such as blood viscosity.

Zajac et al. assessed TKE [32] as a measure of flow inefficiency. They found peak TKE at late diastolic filling was higher in DCM patients compared to healthy volunteers (3.0 ± 1.8 mJ vs 1.5 ± 0.8 mJ, *P* = 0.02). This supports earlier findings by Bolger et al. demonstrating the reduced efficiency of DCM hearts [42]. There is conflicting evidence regarding vortex characteristics in LV dysfunction [24], [36]. Toger et al. found a smaller proportion of blood volume is incorporated within vortices of these patients compared to healthy volunteers [36]. However, this is inconsistent with findings of increased sphericity, greater transverse lengths and larger area for LV vortices in patients with impaired LV function [24]. These variances could be due to chance given the small samples or the differences in how the patients' clinical features were defined.

In a study investigating flow changes in hypertrophic cardiomyopathy, patients with increased extracellular volume (ECV), from fibrosis, had greater energy losses (*P* < 0.001) and elevated pressure gradients (*P* < 0.001) at the LV outflow tract [26].

### 4.2 Heart valves

#### 4.2.1 Mitral valve

Westenberg et al. validated the use of retrospective valve tracking for valvular flow quantification through comparisons with conventional CMR [47]. Retrospective valve tracking was used to accurately and reliably quantify flow, showing strong correlations with aortic systolic stroke volume reference standard (*r* = 0.96, *P* < 0.01 for MV flow). Roes et al. investigated the intra −/inter-observer variability of net flow volumes using retrospective valve tracking [46]. For the MV of healthy volunteers, retrospective valve tracking showed excellent agreement (intraclass correlation coefficient [ICC] = 0.97, *P* < 0.001 and ICC = 0.91, *P* < 0.001 for intra-/inter-observer variability respectively). Similar levels of agreement were demonstrated for patients with valvular regurgitation.

#### 4.2.2 Aortic valve

Retrospective valve tracking is also reliable for net flow quantification across the aortic valve (AV), with an ICC of 0.93, *P* < 0.001 for intra-observer results, and an ICC of 0.98, *P* < 0.001 for inter-observer findings [46]. In a larger study, 4D flow CMR had a sensitivity and specificity of 100% and 98% respectively for identifying aortic regurgitation (AR) [50]. In a study grading AR severity, 2D echocardiography showed limited correlation with 4D flow (κ = 0.53) [52]. The authors speculate that these differences are the result of the assumptions 2D echocardiography requires for its calculation of AR, such as eccentric jets and noncircular orifices.

The only study evaluating aortic stenosis (AS) proposed using the jet shear layer detection (JSLD) method to calculate the AV's effective orifice area (EOA) [51]. Not only did 4D flow CMR show a strong agreement with current 2D EOA methods (*r* = 0.91, *P* < 0.001), but it allows for improvements in identifying the true position of the vena contracta, reducing sources of measurement error.

#### 4.2.3 Tricuspid valve

Two studies have demonstrated the robustness of quantifying tricuspid valve (TV) flow using retrospective valve tracking, against the aortic systolic stroke volume reference standard (*r* = 0.88, *P* < 0.01) [47] and with strong intra-/inter-observer agreement (ICC = 0.93, *P* < 0.001 and ICC = 0.94, *P* < 0.01 respectively) [46].

#### 4.2.4 Pulmonary valve

One study investigated pulmonary valve (PV) flow patterns using retrospective valve tracking [46]. This technique is highly repeatable for PV flow quantification in those with and without valvular regurgitation (intra-/inter-observer variability, ICC = 0.99 and 0.95, *P* < 0.001 respectively). The lack of studies investigating this structure could be due to the reduced incidence of PV pathology with respect to other valvular diseases [56].

### 4.3 Right heart

#### 4.3.1 Right atrium

Arvidsson et al. suggested that the conversion of rotational flow into helical flow in the right atrium (RA) may be a method of conserving atrial KE during right ventricular (RV) filling [18]. This is supplemented by findings that 79% of RA stroke volume is comprised of a single vortex [53], with further evidence of helical flow existing between the RA and the RV [16].

#### 4.3.2 Right ventricle

Several studies have shown RV haemodynamics are different to that of the LV [16], [35], [55]. One study found that RV ‘Direct flow’ was larger and made up a greater proportion of the blood volume at end-diastole, compared to LV ‘Direct flow’ (*P* < 0.01) [55]. It is thought that these conditions, specific to the RV, allow for maximal systolic ejection efficiency of the ‘Direct flow’ volume. This is consistent with findings of higher systolic RV KE compared to the LV (*P* = 0.004) [35].

Additional KE research found the percentage of viscous dissipation was significantly larger in pulmonary arterial hypertension (PAH) patients compared to healthy volunteers (21.1 ± 6.4% vs 2.2 ± 3.1%, *P* = 0.0001, ICC = 0.995) [54]. PAH patients also displayed a greater RV KE work density (94.7 ± 33.7 mJ/ml vs 61.7 ± 14.8 mJ/ml, *P* = 0.007, ICC = 0.990).

## 5 Clinical perspective

Current technological advances in acceleration methods have enabled 4D flow to be acquired in < 10 min, with a reasonable spatial and temporal resolution. Given these optimisations, it is feasible to incorporate 4D flow acquisition within current clinical CMR protocols. The results from this systematic review suggest that retrospective valve tracking for valvular flow quantification, velocity profiling/KE for various chambers of the heart and several visualisation techniques have the most clinical applicability.

**Fig. 3:**
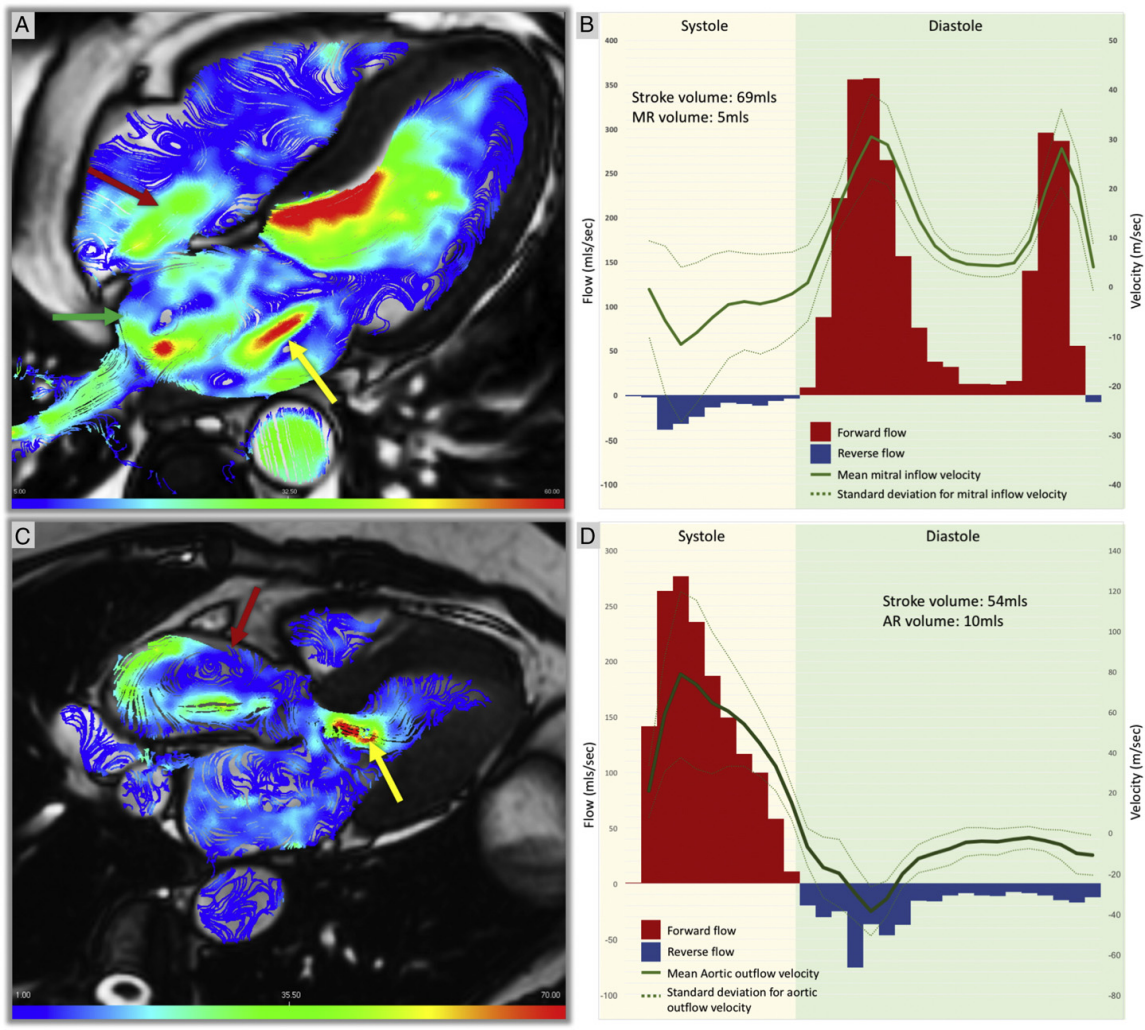
4D flow streamline visualisation and retrospective valve tracking quantification. Panels A and B = Four-dimensional mitral inflow in a patient with mitral regurgitation. Panel A shows the mitral regurgitation (yellow arrow) as well as tricuspid regurgitation (red arrow). Panel B = Mitral valve inflow quantification using retrospective valve-tracking. Panels C and D = Four-dimensional aortic flow in a patient with aortic root dilatation. Panel C shows pathological vortex formation in the ascending aorta (red arrow) as well as aortic regurgitation (yellow arrow). Panel D = Aortic valve flow quantification using retrospective valve-tracking.

Firstly, retrospective valve tracking is a highly reproducible and accurate 4D flow method (Fig. 3). Moreover, it now features within commercial software packages, for example CAAS by PIE Medical Imaging, Maastricht, The Netherlands. This facilitates its incorporation into clinical studies and increases its availability for routine use. Retrospective valve tracking circumvents issues seen with 2D imaging, chiefly through-plane motion errors when quantifying mitral inflow velocities. Despite the enhanced reliability of these measures, the added value that this may bring to diastolic assessments is yet to be validated clinically. Velocity measurements derived by 4D flow CMR have shown similar correlation to velocities calculated using transesophageal echocardiography (TOE) [15]. Compared to TOE, 4D flow is non-invasive and so, is a more tolerable investigation. It can also be used in patients with contraindications to TOE and does not carry the inherent risks associated with anaesthesia and intubation. Furthermore, velocity/KE profiles have characterised unique flow differences between health and disease. Given the complex classification and management of many cardiac pathologies, it is likely that these 4D flow methods can provide new parameters in which to risk stratify these patients, prioritising targeted preventative interventions. Finally, visualisation is a key strength of 4D flow. It is a versatile technique, including vector graphs, streamlines and pathline/particle tracing. Visualisation provides detailed flow maps which can aid in making distinct assessments of haemodynamic disturbances, including alterations to vortex architecture, to supplement velocity and KE findings. However, it is critical that the presentation of this visualisation data is standardised.

## 6 Limitations

Systematic reviews can only synthesise existing literature, therefore any biases or limitations in the included studies will reduce the reliability of the results included for review. The studies reviewed showed considerable methodological heterogeneity, which may have affected inter-study comparisons. For this reason, meta-analyses were not performed. The aspects of subjectivity within the modified CASP tool were minimised through the use of various prompts for each scoring component. Assessing authors have 3 years of 4D flow experience, which may have influenced the results. Nevertheless, the culmination of over 10 years' experience in cardiovascular medicine, enables a qualified assessment of both study quality and the clinical feasibility of developing methods.

## 7 Conclusion

Current literature in 4D flow CMR is mainly single-centred and mechanistic. The available data offer novel insights into intra-cardiac flow patterns, their possible clinical relevance and demonstrate that 4D flow CMR is a precise and reliable tool for flow quantification. The developed methods with the most clinical applicability appear to be retrospective valve tracking, velocity profiling/KE and visualisation techniques. Prospective, randomised, multi-centred studies are required to investigate the incremental benefits 4D flow CMR may offer over standard practices.

A list of abbreviations and definitions is provided in the supplemental file.

## Competing interests

None.

## Appendix A Supplementary data

Supplementary file Image 1
