# Supplementary material for: Clinical applications of intra-cardiac four-dimensional flow cardiovascular magnetic resonance: A systematic review
Source: Int J Cardiol. 2017 Dec 15;249:486–93. doi: 10.1016/j.ijcard.2017.07.023 (PMC5687937; doi:10.1016/j.ijcard.2017.07.023)
Supplement: Supplementary file 1 — Supplementary file [file mmc1.docx]

**SUPPLEMENTAL FILE**

*Clinical applications of Intra-cardiac Four-dimensional Flow Cardiovascular Magnetic Resonance: A Systematic Review*

Table of Contents

[Search strategy 2](#_Toc472875700)

[Quality assessment 3](#_Toc472875701)

[Results 3](#_Toc472875702)

[Figures 4](#_Toc472875703)

[Tables 7](#_Toc472875704)

[References 16](#_Toc472875705)

## Search strategy

The following search strategy was used on: Medline, EMBASE, Cochrane Library, Copac, OpenGrey and clinicaltrials.gov databases.

As four-dimensional flow cardiovascular magnetic resonance (4D flow CMR) is a novel, limited research area, all articles using 4D flow CMR were initially sought after.

The search for all 4D flow studies was split into two separate searches. This is following the recommendations from the 4D flow CMR consensus document regarding standardised terminology^1^. The consensus document recommends using “4D flow CMR” or “4D flow MRI” or the full term, “three-dimensional (3D) cine (time-resolved) phase-contrast (PC) CMR with three-directional velocity-encoding”. As such, these terms can be broken down as follows (see Figure 1):

As a result, the first search was as follows:

1. ((4D) OR (four-dimensional)) AND (flow)
2. ((3D) OR (three-dimensional)) AND ((cine) OR (time-resolved)) AND ((PC) OR (phase contrast))
3. 1. OR 2.
4. ((CMR) OR (cardiovascular magnetic resonance) OR (MRI) OR (magnetic resonance imaging))
5. 3. AND 4.
6. Limit to English and Humans only

A full breakdown of the first search can be seen in Figure 2 below.

It was found that adding the term “velocity-encod*” yielded only 3 results. To minimise selection bias, a second search was done to capture articles that may have used these terms.

* = truncation, which allows for all alternate endings of velocity-encod- to be searched for i.e. ‘velocity encoded’ as well as ‘velocity encoding’

The second search was as follows:

1. ((3D) OR (three-dimensional) OR (three-directional))
2. Velocity encod*
3. 1. AND 2.
4. Limit to English and Humans only

A full breakdown of the second search can be seen in Figure 3 below.

## Quality assessment

The modified Critical Appraisal Skills Programme (CASP) tool is shown in full in Table 1 below. The scoring tool was developed by combining questions from various CASP checklists^2^ that were applicable to the included studies, as well as modifying three questions to increase their relevance.

## Results

The full breakdown of scores for each of the included studies is shown in Table 3.

All relevant conference proceedings/abstracts are detailed in Table 4.

**Figures**

**Figure 1.** *Diagram representing the term, four-dimensional flow cardiovascular magnetic resonance, was broken down for the search strategy. The 4D flow consensus document recommendations^1^ were the basis of the terms used.*

4D/ four-dimensional AND flow

CMR/ cardiovascular magnetic resonance

3D/ three-dimensional cine/ time-resolved
PC/ phase-contrast
velocity-encoded

MRI/ magnetic resonance imaging

AND

OR

OR

**Figure 2.** *Screenshot example of the first search used for database searching. This example is taken from Ovid Medline.*


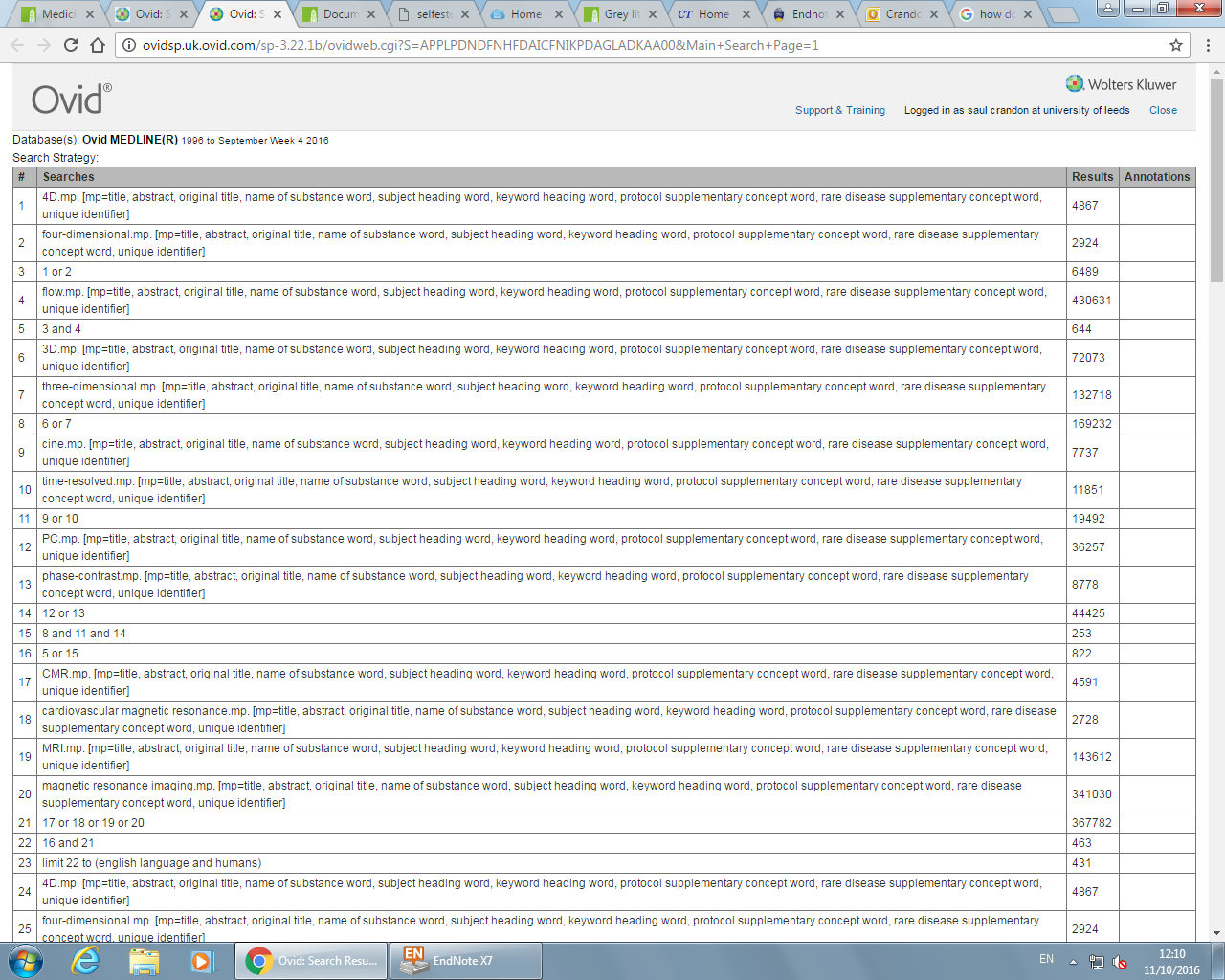


**Figure 3.** *Screenshot example of the second search used for database searching. This example is taken from Ovid Medline.*


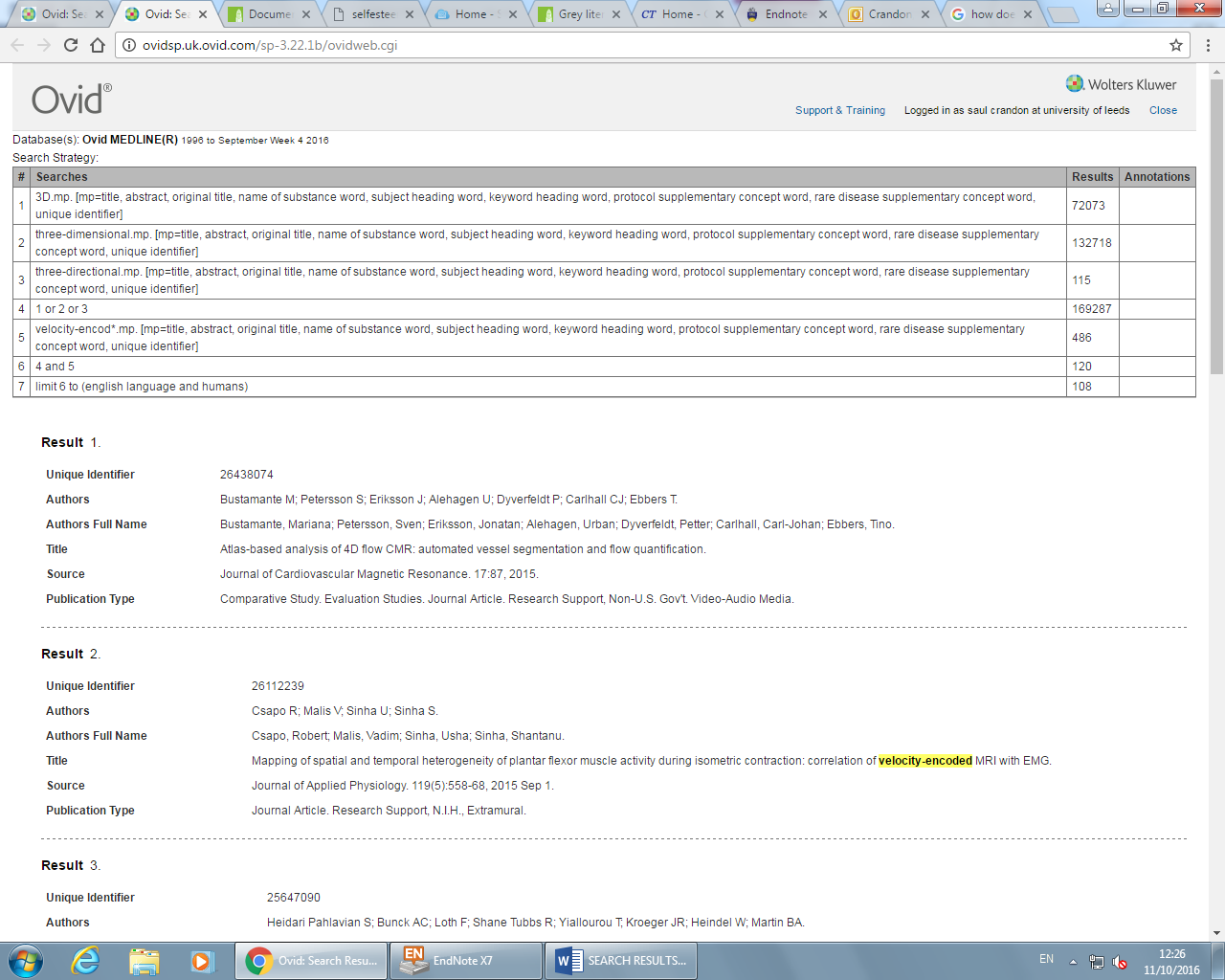


## Tables

**Table 1.** *Modified Critical Skills Appraisal Programme (CASP) scoring tool. An answer of ‘yes’ scores 1 point, an answer of ‘no’ or ‘can’t tell’ scores 0 points. This tool was used to score the included studies and rank them in order of clinical applicability. The tool was modified to increase its relevance to the included studies. The CASP checklists are distributed under the terms of the Creative Commons Attribution License, which permits unrestricted use, distribution, and reproduction in any medium.
ᵃ Original CASP checklist questions
ᵇ Modified CASP checklist questions*

| **Full version of the modified CASP scoring tool** | |
| --- | --- |
| **Question** | **Answer** |
| **1. Is the disease status of the tested population clearly defined? ᵃ** *-Did they have a clear diagnosis? -Was the disease severity stated?* | Yes/No/Can’t Tell |
| **2. Were the cases recruited in an acceptable way? ᵃ**  *-Were the cases representative of a defined population? (external validity) -Was there an established reliable system for selecting all the cases? -Was there a sufficient number of cases selected? -Was a power calculation included?* | Yes/No/Can’t Tell |
| **3. Were the controls recruited in an acceptable way? ᵃ**  *-Were the controls representative of a defined population? (external validity) -Was there a sufficient number of controls selected? -Are they matched, population-based or randomly selected?* | Yes/No/Can’t Tell |
| **4. Have the authors identified all important confounding factors? ᵃ**  *-Race, age, sex, gender, ethnicity, health status, socioeconomic status etc.* | Yes/No/Can’t Tell |
| **5. Have the authors taken account of these confounding factors in the design and/or analysis? ᵃ** *-Design restrictions and techniques e.g. modelling, stratified-, regression-, sensitivity analysis to correct, control or adjust for confounding factors* | Yes/No/Can’t Tell |
| **6. Were the methods for performing the test described in sufficient detail? ᵃ** *-Was a protocol followed?* | Yes/No/Can’t Tell |
| **7. Are the results clear? ᵇ** | Yes/No/Can’t Tell |
| **8. Are the results precise? ᵇ** *-Size of the confidence intervals (95%) -Size of the p value (0.05) -Are all important variables considered?* | Yes/No/Can’t Tell |
| **9. Are the results reliable? ᵇ** *-Could the results be due to bias, chance or confounding? -Is the inter- and/ or intraobserver variability provided?* | Yes/No/Can’t Tell |
| **10. Can the results be applied locally/ to your patients/ to your population of interest? ᵃ** *-Is the study population similar enough to your population that you can apply the results even if your population may fall outside the study’s inclusion criteria?* | Yes/No/Can’t Tell |
| **11. Can the test be applied locally/ to your patients/ to your population of interest? ᵃ** *-Resources and opportunity costs  -Level and availability of expertise required to interpret the tests -Current practice and availability of services* | Yes/No/Can’t Tell |
| **12. Do the results of the study fit with the other available evidence? ᵃ** | Yes/No/Can’t Tell |
| **13. Were all outcomes important to the individual or population considered? ᵃ** | Yes/No/Can’t Tell |

**Table 2.** *Supplemental table of scores assigned to the 44 included studies.* *The scores obtained on the modified CASP tool, along with percentages and subsequent clinical applicability categorisation is shown. Scores of 67-100% were considered ‘highly clinical applicable’, 34-66% considered ‘potentially clinical applicable’ and 0-33% considered ‘less clinically applicable’ for the purposes of this review.*

| **Scoring results of the included studies** | | | | | | | | | | | | | | | | |
| --- | --- | --- | --- | --- | --- | --- | --- | --- | --- | --- | --- | --- | --- | --- | --- | --- |
| **Questions on the modified CASP tool** | | | | | | | | | | | | | | | | |
| **Author(s), year** | **1** | **2** | **3** | **4** | **5** | **6** | **7** | **8** | **9** | **10** | **11** | **12** | **13** | **Total score** | **%** | **Clinically applicable** |
| Al-Wakeel et al, 2015 ^3^ | 1 | 0 | 0 | 0 | 1 | 0 | 1 | 1 | 1 | 1 | 1 | 1 | 0 | 8 | 62 | Potentially |
| Arvidsson et al, 2013 ^4^ | 0 | 1 | 1 | 1 | 0 | 1 | 1 | 1 | 1 | 0 | 1 | 1 | 1 | 10 | 77 | Highly |
| Bolger et al, 2007 ^5^ | 0 | 0 | 1 | 0 | 0 | 1 | 1 | 0 | 1 | 0 | 0 | 1 | 0 | 5 | 38 | Potentially |
| Brandts et al, 2011 ^6^ | 1 | 1 | 0 | 0 | 0 | 1 | 1 | 1 | 1 | 1 | 1 | 1 | 1 | 10 | 77 | Highly |
| Callaghan et al, 2016 ^7^ | 0 | 1 | 1 | 1 | 0 | 1 | 1 | 1 | 1 | 1 | 1 | 1 | 1 | 11 | 85 | Highly |
| Carlsson et al, 2012 ^8^ | 0 | 1 | 1 | 0 | 0 | 1 | 1 | 0 | 1 | 0 | 1 | 1 | 0 | 7 | 54 | Potentially |
| Chelu et al, 2016 ^9^ | 1 | 1 | 0 | 0 | 1 | 1 | 1 | 1 | 1 | 1 | 1 | 1 | 1 | 11 | 85 | Highly |
| Dyverfeldt et al, 2011 ^10^ | 1 | 0 | 0 | 0 | 0 | 0 | 1 | 1 | 1 | 0 | 0 | 1 | 0 | 5 | 38 | Potentially |
| Ebbers et al, 2002 ^11^ | 0 | 0 | 0 | 0 | 0 | 1 | 1 | 0 | 0 | 0 | 0 | 0 | 0 | 2 | 15 | Less |
| Elbaz et al, 2014 ^12^ | 1 | 1 | 1 | 0 | 0 | 1 | 1 | 1 | 1 | 1 | 0 | 1 | 0 | 9 | 69 | Highly |
| Eriksson et al, 2016 ^13^ | 1 | 1 | 1 | 1 | 1 | 1 | 1 | 0 | 0 | 0 | 0 | 1 | 0 | 8 | 62 | Potentially |
| Eriksson et al, 2013 ^14^ | 1 | 1 | 1 | 1 | 1 | 1 | 1 | 1 | 1 | 1 | 1 | 1 | 1 | 13 | 100 | Highly |
| Eriksson et al, 2011 ^15^ | 0 | 0 | 1 | 0 | 0 | 1 | 1 | 0 | 0 | 0 | 0 | 0 | 0 | 3 | 23 | Less |
| Eriksson et al, 2010 ^16^ | 1 | 1 | 1 | 0 | 0 | 1 | 1 | 0 | 1 | 1 | 1 | 1 | 1 | 10 | 77 | Highly |
| Eriksson et al, 2015 ^17^ | 1 | 1 | 1 | 0 | 0 | 1 | 1 | 1 | 0 | 0 | 0 | 1 | 0 | 7 | 54 | Potentially |
| Ewe et al, 2013 ^18^ | 1 | 1 | 1 | 0 | 0 | 1 | 1 | 1 | 1 | 1 | 1 | 1 | 1 | 11 | 85 | Highly |
| Fluckiger et al, 2013 ^19^ | 1 | 1 | 0 | 0 | 0 | 1 | 0 | 0 | 0 | 0 | 0 | 1 | 0 | 4 | 31 | Less |
| Foll et al, 2013 ^20^ | 1 | 0 | 0 | 1 | 0 | 1 | 1 | 1 | 1 | 0 | 0 | 1 | 0 | 7 | 54 | Potentially |
| Fredriksson et al, 2011 ^21^ | 1 | 1 | 1 | 0 | 0 | 1 | 1 | 1 | 1 | 0 | 0 | 1 | 0 | 8 | 62 | Potentially |
| Fyrenius et al, 2001 ^22^ | 1 | 1 | 1 | 0 | 0 | 1 | 1 | 1 | 0 | 1 | 1 | 1 | 1 | 10 | 77 | Highly |
| Garcia et al, 2014 ^23^ | 1 | 1 | 1 | 1 | 1 | 1 | 1 | 1 | 1 | 1 | 1 | 1 | 1 | 13 | 100 | Highly |
| Han et al, 2015 ^24^ | 1 | 1 | 0 | 0 | 0 | 1 | 0 | 0 | 0 | 0 | 0 | 0 | 0 | 3 | 23 | Less |
| Kanski et al, 2015 ^25^ | 1 | 1 | 0 | 0 | 0 | 1 | 1 | 1 | 1 | 1 | 1 | 1 | 1 | 10 | 77 | Highly |
| Kanski et al, 2015 ^26^ | 1 | 1 | 1 | 0 | 0 | 1 | 1 | 1 | 1 | 1 | 1 | 1 | 1 | 11 | 85 | Highly |
| Kim et al, 1995 ^27^ | 1 | 1 | 1 | 0 | 0 | 1 | 1 | 1 | 0 | 1 | 1 | 1 | 1 | 10 | 77 | Highly |
| Kumar et al, 2011 ^28^ | 1 | 0 | 0 | 0 | 0 | 0 | 1 | 0 | 0 | 0 | 0 | 1 | 0 | 3 | 23 | Less |
| Lee et al, 2016 ^29^ | 1 | 1 | 1 | 1 | 1 | 1 | 1 | 1 | 0 | 1 | 1 | 1 | 1 | 12 | 92 | Highly |
| Markl et al, 2016 ^30^ | 1 | 1 | 1 | 1 | 1 | 1 | 1 | 1 | 0 | 1 | 1 | 1 | 1 | 12 | 92 | Highly |
| Markl et al, 2016 ^31^ | 1 | 1 | 1 | 1 | 1 | 1 | 1 | 1 | 0 | 1 | 1 | 1 | 1 | 12 | 92 | Highly |
| Markl et al, 2016 ^32^ | 1 | 1 | 1 | 1 | 1 | 1 | 1 | 1 | 0 | 1 | 1 | 1 | 1 | 12 | 92 | Highly |
| Marsan et al, 2009 ^33^ | 1 | 1 | 1 | 0 | 0 | 1 | 1 | 0 | 1 | 1 | 1 | 1 | 1 | 10 | 77 | Highly |
| Roes et al, 2009 ^34^ | 1 | 1 | 1 | 0 | 0 | 1 | 1 | 0 | 1 | 1 | 1 | 1 | 1 | 10 | 77 | Highly |
| Steding-Ehrenborg et al, 2015 ^35^ | 1 | 1 | 0 | 0 | 0 | 1 | 1 | 0 | 1 | 1 | 1 | 1 | 1 | 9 | 69 | Highly |
| Suwa et al, 2015 ^36^ | 1 | 1 | 0 | 1 | 1 | 1 | 1 | 0 | 1 | 1 | 1 | 1 | 1 | 11 | 85 | Highly |
| Suwa et al, 2016 ^37^ | 1 | 0 | 0 | 0 | 0 | 1 | 1 | 1 | 1 | 1 | 1 | 1 | 1 | 9 | 69 | Highly |
| Svalbring et al, 2016 ^38^ | 1 | 0 | 0 | 0 | 0 | 1 | 1 | 0 | 0 | 1 | 1 | 1 | 0 | 6 | 46 | Potentially |
| Toger et al, 2011 ^39^ | 1 | 0 | 0 | 0 | 0 | 0 | 0 | 0 | 0 | 0 | 0 | 1 | 0 | 2 | 15 | Less |
| Toger et al, 2012 ^40^ | 1 | 0 | 0 | 0 | 0 | 1 | 0 | 0 | 0 | 0 | 0 | 1 | 0 | 3 | 23 | Less |
| Van Ooij et al, 2016 ^41^ | 1 | 0 | 0 | 0 | 0 | 1 | 1 | 1 | 1 | 1 | 1 | 1 | 1 | 9 | 69 | Highly |
| Westenberg et al, 2004 ^42^ | 1 | 1 | 0 | 0 | 1 | 1 | 1 | 1 | 1 | 1 | 1 | 1 | 1 | 11 | 85 | Highly |
| Westenberg et al, 2005 ^43^ | 1 | 1 | 0 | 0 | 0 | 1 | 1 | 1 | 0 | 1 | 1 | 1 | 1 | 9 | 69 | Highly |
| Westenberg et al, 2008 ^44^ | 1 | 1 | 1 | 1 | 1 | 1 | 1 | 1 | 1 | 1 | 1 | 1 | 1 | 13 | 100 | Highly |
| Wong et al, 2016 ^45^ | 1 | 1 | 1 | 0 | 0 | 1 | 1 | 1 | 0 | 1 | 1 | 1 | 1 | 10 | 77 | Highly |
| Zajac et al, 2014 ^46^ | 1 | 0 | 0 | 0 | 0 | 1 | 1 | 1 | 0 | 0 | 0 | 0 | 0 | 4 | 31 | Less |

**Table 3.** *Table of definitions used to label the included studies. These were predetermined by the authors of this review. Studies that stated their own study type were labelled according to these statements. Studies were also labelled as ‘prospective’ or ‘retrospective’ if these terms were explicitly stated in the article. 4D flow CMR=four dimensional flow cardiovascular magnetic resonance.*

| **Study type** | **Definition** |
| --- | --- |
| Mechanistic | A study that examines a mechanism of intra-cardiac flow in health and/ or disease. |
| Pilot | A study that assesses the feasibility and/ or provides initial data for a proposed method of intra-cardiac quantification using 4D flow CMR. |
| Diagnostic | A study that assesses a method’s diagnostic capabilities, with comparison to a reference standard. |

**Table 4.** *The relevant 29 abstracts are categorised according to intra-cardiac structure and presented in reverse chronological order. Abstracts that are relevant to more than one intra-cardiac structure are denoted by an asterisk (*).*

| Intra-cardiac subcategory | Abstracts |
| --- | --- |
| Left Atrium | *Mark et al 2016* ^47^  *Steding-Ehrenborg et al 2015** ^48^  *Carr et al 2014** ^49^*,* *Contaldi et al 2014* ^50^, *Lee et al 2014* ^51^  *Ng et al 2014* ^52^  *Fluckiger et al 2013* ^53^*,* *Goldberger et al 2013* ^54^*,* *Lee et al 2013* ^55^*,* *Saitoh et al 2013** ^56^ |
| Left Ventricle | *Erixon et al 2016* ^57^, *Saru et al 2016* ^58^, *Stoll et al 2016* ^59^,  *Toger et al 2016* ^60^*, Zajac et al 2016* ^61^  *Hussaini et al 2015* ^62^*, Steding-Ehrenborg et al 2015** ^48^, *Stoll et al 2015* ^63^  *Chu et al 2014* ^64^*, Elbaz et al 2014** ^65^*, Zhang et al 2014** ^66^  *Saitoh et al 2013** ^56^*, Zajac et al 2013* ^67^  *Eriksson et al 2012* ^68^ |
| Mitral Valve | *Garg et al 2016** ^69^ *Lessick et al 2014* ^70^*, Zhang et al 2014** ^66^ |
| Aortic Valve | *Garg et al 2016** ^69^*, Nordmeyer et al 2013** ^71^ |
| Tricuspid Valve | *Roldan-Alzate et al 2013* ^72^ |
| Pulmonary Valve | *Nordmeyer et al 2013** ^71^ |
| Right Atrium | *Steding-Ehrenborg et al 2015** ^48^  *Carr et al 2014** ^49^ |
| Right Ventricle | *Fredriksson et al 2016* ^73^  *Hussaini et al 2015* ^62^*, Steding-Ehrenborg et al 2015** ^48^  *Elbaz et al 2014** ^65^*, Fenster et al 2013* ^74^*, Kheyfets et al 2014* ^75^ |

**Table 5.** *Abbreviations and definitions of the terms used throughout the review.*

| **Table of abbreviations and definitions** | |
| --- | --- |
| **2D** | **Two-dimensional** |
| **3D** | **Three-dimensional** |
| **4D** | **Four-dimensional** |
| **AF** | **Atrial fibrillation** – an arrhythmia in the atria that can increase stroke risk |
| **ALVA** | **Apical left ventricular aneurysm** – aneurysm at the apex of the left ventricle |
| **AR** | **Aortic regurgitation** – abnormal, retrograde blood flow through the aortic valve |
| **AS** | **Aortic stenosis** – abnormal narrowing of the aortic valve |
| **AV** | **Aortic valve** |
| **CC** | **Correlation coefficient** – a number that quantifies a correlation |
| **CHA_2_DS_2_-VASc** | An epidemiologically-based scoring system used to assess future stroke risk in AF patients |
| **CMR** | **Cardiovascular magnetic resonance** |
| **DCM** | **Dilated cardiomyopathy** – the heart is enlarged and weakened, failing to pump correctly |
| **DD** | **Diastolic dysfunction** – the ventricles fail to relax normally and can stiffen |
| **Direct flow** | The volume of blood that passes from the atrium to the outflow tract in one heartbeat |
| **E/A** | **Mitral inflow ratio** – ratio of early (E) and late (A) mitral inflow velocities |
| **ECV** | E**xtracellular volume** – the volume of fluid outside cells |
| **EF** | **Ejection fraction** – percentage of blood ejected in one heartbeat |
| **EOA** | **Effective orifice area** – the minimal cross-sectional area of the ejected jet from the aortic valve. A standard parameter in the assessment of the severity of aortic stenosis |
| **HCM** | **Hypertrophic cardiomyopathy** – an enlargement of the heart muscle, that can obstruct the outflow of blood from the heart |
| **HF** | **Heart failure** – an inability of the heart to function correctly |
| **HV** | **Healthy volunteer** |
| **IC** | **Ischaemic cardiomyopathy** – narrowing of the arteries that supply the heart causing a reduction in heart function |
| **ICC** | **Intraclass correlation coefficient** – describes how strongly numbers in a group resemble each other. Can be used to assess inter- and intraobserver variability |
| **IHD** | **Ischaemic heart disease** – a spectrum of disease characterised by narrowing of the arteries that supply the heart |
| **JSLD** | **Jet shear layer detection** – a proposed method of quantifying EOA using 4D flow CMR |
| **KE** | **Kinetic energy** – energy in the movement of a body with mass (KE=0.5mv^2^) |
| **LA** | **Left atrium/ left atrial** |
| **LV** | **Left ventricle/ left ventricular** |
| **LVOT** | **Left ventricular outflow tract** – an area of the heart in which blood passes from the left ventricle into the aorta |
| **MR** | **Mitral regurgitation** – abnormal, retrograde blood flow through the mitral valve |
| **MRI** | **Magnetic resonance imaging** |
| **MV** | **Mitral valve** |
| **NYHA** | **New York Heart Association** |
| **OHD** | **Organic heart disease** – heart disease, in which the problem is within the heart itself |
| **PAH** | **Pulmonary arterial hypertension** – increased blood pressure in the pulmonary arterial system |
| **pEF** | **Heart failure with preserved ejection fraction** |
| **PC** | **Phase contrast** – an MRI technique used to determine flow velocities |
| **PV** | **Pulmonary valve** |
| **RA** | **Right atrium/ right atrial** |
| **rEF** | **Heart failure with reduced ejection fraction** |
| **RV** | **Right ventricle/ right ventricular** |
| **Serum NT-proBNP** | **Serum N-terminal pro-brain natriuretic peptide** – marker of heart failure |
| **TKE** | **Turbulent kinetic energy** – mean KE per unit mass associated with eddies |
| **TR** | **Tricuspid regurgitation** – abnormal, retrograde blood flow through the tricuspid valve |
| **TV** | **Tricuspid valve** |
| **Vena contracta** | The point in a stream of fluid whereby the diameter of the stream is at its minimum, and its velocity is at its maximum. |

## References

1. Dyverfeldt P, Bissell M, Barker AJ, Bolger AF, Carlhall CJ, Ebbers T, Francios CJ, Frydrychowicz A, Geiger J, Giese D, Hope MD, Kilner PJ, Kozerke S, Myerson S, Neubauer S, Wieben O and Markl M. 4D flow cardiovascular magnetic resonance consensus statement. *J Cardiovasc Magn Reson*. 2015;17:72.

2. Critical Skills Appraisal Programme (CASP). CASP Checklists. 2014.

3. Al-Wakeel N, Fernandes JF, Amiri A, Siniawski H, Goubergrits L, Berger F and Kuehne T. Hemodynamic and energetic aspects of the left ventricle in patients with mitral regurgitation before and after mitral valve surgery. *Journal of Magnetic Resonance Imaging*. 2015;42:1705-1712.

4. Arvidsson PM, Toger J, Heiberg E, Carlsson M and Arheden H. Quantification of left and right atrial kinetic energy using four-dimensional intracardiac magnetic resonance imaging flow measurements. *Journal of Applied Physiology*. 2013;114:1472-81.

5. Bolger AF, Heiberg E, Karlsson M, Wigstrom L, Engvall J, Sigfridsson A, Ebbers T, Kvitting JP, Carlhall CJ and Wranne B. Transit of blood flow through the human left ventricle mapped by cardiovascular magnetic resonance. *Journal of Cardiovascular Magnetic Resonance*. 2007;9:741-7.

6. Brandts A, Bertini M, van Dijk EJ, Delgado V, Marsan NA, van der Geest RJ, Siebelink HM, de Roos A, Bax JJ and Westenberg JJ. Left ventricular diastolic function assessment from three-dimensional three-directional velocity-encoded MRI with retrospective valve tracking. *Journal of Magnetic Resonance Imaging*. 2011;33:312-9.

7. Callaghan FM, Arnott C, Figtree GA, Kutty S, Celermajer DS and Grieve SM. Quantifying right atrial filling and emptying: A 4D-flow MRI study. *Journal of Magnetic Resonance Imaging*. 2016.

8. Carlsson M, Heiberg E, Toger J and Arheden H. Quantification of left and right ventricular kinetic energy using four-dimensional intracardiac magnetic resonance imaging flow measurements. *American Journal of Physiology - Heart & Circulatory Physiology*. 2012;302:H893-900.

9. Chelu RG, van den Bosch AE, van Kranenburg M, Hsiao A, van den Hoven AT, Ouhlous M, Budde RPJ, Beniest KM, Swart LE, Coenen A, Lubbers MM, Wielopolski PA, Vasanawala SS, Roos-Hesselink JW and Nieman K. Qualitative grading of aortic regurgitation: a pilot study comparing CMR 4D flow and echocardiography. *International Journal of Cardiovascular Imaging*. 2016;32:301-307.

10. Dyverfeldt P, Kvitting JP, Carlhall CJ, Boano G, Sigfridsson A, Hermansson U, Bolger AF, Engvall J and Ebbers T. Hemodynamic aspects of mitral regurgitation assessed by generalized phase-contrast MRI. *Journal of Magnetic Resonance Imaging*. 2011;33:582-8.

11. Ebbers T, Wigstrom L, Bolger AF, Wranne B and Karlsson M. Noninvasive measurement of time-varying three-dimensional relative pressure fields within the human heart. *Journal of Biomechanical Engineering*. 2002;124:288-93.

12. Elbaz MS, Calkoen EE, Westenberg JJ, Lelieveldt BP, Roest AA and van der Geest RJ. Vortex flow during early and late left ventricular filling in normal subjects: quantitative characterization using retrospectively-gated 4D flow cardiovascular magnetic resonance and three-dimensional vortex core analysis. *Journal of Cardiovascular Magnetic Resonance*. 2014;16:78.

13. Eriksson J, Bolger AF, Ebbers T and Carlhall CJ. Assessment of left ventricular hemodynamic forces in healthy subjects and patients with dilated cardiomyopathy using 4D flow MRI. *Physiological Reports*. 2016;4.

14. Eriksson J, Bolger AF, Ebbers T and Carlhall CJ. Four-dimensional blood flow-specific markers of LV dysfunction in dilated cardiomyopathy. *European Heart Journal - Cardiovascular Imaging*. 2013;14:417-24.

15. Eriksson J, Dyverfeldt P, Engvall J, Bolger AF, Ebbers T and Carlhall CJ. Quantification of presystolic blood flow organization and energetics in the human left ventricle. *American Journal of Physiology - Heart and Circulatory Physiology*. 2011;300:H2135-H2141.

16. Eriksson J, Carlhall CJ, Dyverfeldt P, Engvall J, Bolger AF and Ebbers T. Semi-automatic quantification of 4D left ventricular blood flow. *Journal of Cardiovascular Magnetic Resonance*. 2010;12:9.

17. Eriksson J, Bolger AF, Carlhall CJ and Ebbers T. Spatial heterogeneity of four-dimensional relative pressure fields in the human left ventricle. *Magn Reson Med*. 2015;74:1716-25.

18. Ewe SH, Delgado V, van der Geest R, Westenberg JJ, Haeck ML, Witkowski TG, Auger D, Marsan NA, Holman ER, de Roos A, Schalij MJ, Bax JJ, Sieders A and Siebelink HM. Accuracy of three-dimensional versus two-dimensional echocardiography for quantification of aortic regurgitation and validation by three-dimensional three-directional velocity-encoded magnetic resonance imaging. *American Journal of Cardiology*. 2013;112:560-6.

19. Fluckiger JU, Goldberger JJ, Lee DC, Ng J, Lee R, Goyal A and Markl M. Left atrial flow velocity distribution and flow coherence using four-dimensional FLOW MRI: a pilot study investigating the impact of age and Pre- and Postintervention atrial fibrillation on atrial hemodynamics. *Journal of Magnetic Resonance Imaging*. 2013;38:580-7.

20. Foll D, Taeger S, Bode C, Jung B and Markl M. Age, gender, blood pressure, and ventricular geometry influence normal 3D blood flow characteristics in the left heart. *European heart journal cardiovascular Imaging*. 2013;14:366-73.

21. Fredriksson AG, Zajac J, Eriksson J, Dyverfeldt P, Bolger AF, Ebbers T and Carlhall CJ. 4-D blood flow in the human right ventricle. *American Journal of Physiology - Heart & Circulatory Physiology*. 2011;301:H2344-50.

22. Fyrenius A, Wigstrom L, Ebbers T, Karlsson M, Engvall J and Bolger A. Three dimensional flow in the human left atrium. *Heart*. 2001;86:448-455.

23. Garcia J, Markl M, Schnell S, Allen B, Entezari P, Mahadevia R, Chris Malaisrie S, Pibarot P, Carr J and Barker AJ. Evaluation of aortic stenosis severity using 4D flow jet shear layer detection for the measurement of valve effective orifice area. *Magnetic Resonance Imaging*. 2014;32:891-8.

24. Han QJ, Witschey WR, Fang-Yen CM, Arkles JS, Barker AJ, Forfia PR and Han Y. Altered Right Ventricular Kinetic Energy Work Density and Viscous Energy Dissipation in Patients with Pulmonary Arterial Hypertension: A Pilot Study Using 4D Flow MRI. *PLoS ONE [Electronic Resource]*. 2015;10:e0138365.

25. Kanski M, Arvidsson PM, Toger J, Borgquist R, Heiberg E, Carlsson M and Arheden H. Left ventricular fluid kinetic energy time curves in heart failure from cardiovascular magnetic resonance 4D flow data. *Journal of Cardiovascular Magnetic Resonance*. 2015;17:111.

26. Kanski M, Toger J, Steding-Ehrenborg K, Xanthis C, Bloch KM, Heiberg E, Carlsson M and Arheden H. Whole-heart four-dimensional flow can be acquired with preserved quality without respiratory gating, facilitating clinical use: a head-to-head comparison. *BMC Medical Imaging*. 2015;15:20.

27. Kim WY, Walker PG, Pedersen EM, Poulsen JK, Oyre S, Houlind K and Yoganathan AP. Left ventricular blood flow patterns in normal subjects: A quantitative analysis by three-dimensional magnetic resonance velocity mapping. *Journal of the American College of Cardiology*. 1995;26:224-238.

28. Kumar R, Charonko J, Hundley WG, Hamilton CA, Stewart KC, McNeal GR, Vlachos PP and Little WC. Assessment of left ventricular diastolic function using 4-dimensional phase-contrast cardiac magnetic resonance. *Journal of Computer Assisted Tomography*. 2011;35:108-12.

29. Lee DC, Markl M, Ng J, Carr M, Benefield B, Carr JC and Goldberger JJ. Three-dimensional left atrial blood flow characteristics in patients with atrial fibrillation assessed by 4D flow CMR. *European Heart Journal – Cardiovascular Imaging*. 2016;17:1259-1268.

30. Markl M, Carr M, Ng J, Lee DC, Jarvis K, Carr J and Goldberger JJ. Assessment of left and right atrial 3D hemodynamics in patients with atrial fibrillation: a 4D flow MRI study. *International Journal of Cardiovascular Imaging*. 2016;32:807-815.

31. Markl M, Lee DC, Furiasse N, Carr M, Foucar C, Ng J, Carr J and Goldberger JJ. Left Atrial and Left Atrial Appendage 4D Blood Flow Dynamics in Atrial Fibrillation. *Circulation: Cardiovascular Imaging*. 2016;9 (9) (no pagination).

32. Markl M, Lee DC, Ng J, Carr M, Carr J and Goldberger JJ. Left atrial 4-dimensional flow magnetic resonance imaging stasis and velocity mapping in patients with atrial fibrillation. *Investigative Radiology*. 2016;51:147-154.

33. Marsan NA, Westenberg JJ, Ypenburg C, Delgado V, van Bommel RJ, Roes SD, Nucifora G, van der Geest RJ, de Roos A, Reiber JC, Schalij MJ and Bax JJ. Quantification of functional mitral regurgitation by real-time 3D echocardiography: comparison with 3D velocity-encoded cardiac magnetic resonance. *Jacc: Cardiovascular Imaging*. 2009;2:1245-52.

34. Roes SD, Hammer S, van der Geest RJ, Marsan NA, Bax JJ, Lamb HJ, Reiber JH, de Roos A and Westenberg JJ. Flow assessment through four heart valves simultaneously using 3-dimensional 3-directional velocity-encoded magnetic resonance imaging with retrospective valve tracking in healthy volunteers and patients with valvular regurgitation. *Investigative Radiology*. 2009;44:669-75.

35. Steding-Ehrenborg K, Arvidsson PM, Toger J, Rydberg M, Heiberg E, Carlsson M and Arheden H. Determinants of kinetic energy of blood flow in the four-chambered heart in athletes and sedentary controls. *American Journal of Physiology - Heart & Circulatory Physiology*. 2016;310:H113-22.

36. Suwa K, Saitoh T, Takehara Y, Sano M, Nobuhara M, Saotome M, Urushida T, Katoh H, Satoh H, Sugiyama M, Wakayama T, Alley M, Sakahara H and Hayashi H. Characteristics of intra-left atrial flow dynamics and factors affecting formation of the vortex flow - analysis with phase-resolved 3-dimensional cine phase contrast magnetic resonance imaging. *Circulation Journal*. 2015;79:144-52.

37. Suwa K, Saitoh T, Takehara Y, Sano M, Saotome M, Urushida T, Katoh H, Satoh H, Sugiyama M, Wakayama T, Alley M, Sakahara H and Hayashi H. Intra-left ventricular flow dynamics in patients with preserved and impaired left ventricular function: Analysis with 3D cine phase contrast MRI (4D-Flow). *Journal of Magnetic Resonance Imaging*. 2016.

38. Svalbring E, Fredriksson A, Eriksson J, Dyverfeldt P, Ebbers T, Bolger AF, Engvall J and Carlhall CJ. Altered diastolic flow patterns and kinetic energy in subtle left ventricular remodeling and dysfunction detected by 4D flow MRI. *PLoS ONE*. 2016;11 (8) (no pagination).

39. Toger J, Carlsson M, Soderlind G, Arheden H and Heiberg E. Volume Tracking: A new method for quantitative assessment and visualization of intracardiac blood flow from three-dimensional, time-resolved, three-component magnetic resonance velocity mapping. *BMC Medical Imaging*. 2011;11:10.

40. Toger J, Kanski M, Carlsson M, Kovacs SJ, Soderlind G, Arheden H and Heiberg E. Vortex ring formation in the left ventricle of the heart: analysis by 4D flow MRI and Lagrangian coherent structures. *Annals of Biomedical Engineering*. 2012;40:2652-62.

41. Van Ooij P, Allen BD, Contaldi C, Garcia J, Collins J, Carr J, Choudhury L, Bonow RO, Barker AJ and Markl M. 4D flow MRI and T1-Mapping: Assessment of altered cardiac hemodynamics and extracellular volume fraction in hypertrophic cardiomyopathy. *Journal of Magnetic Resonance Imaging*. 2016;43:107-114.

42. Westenberg JJ, Danilouchkine MG, Doornbos J, Bax JJ, van der Geest RJ, Labadie G, Lamb HJ, Versteegh MI, de Roos A and Reiber JH. Accurate and reproducible mitral valvular blood flow measurement with three-directional velocity-encoded magnetic resonance imaging. *Journal of Cardiovascular Magnetic Resonance*. 2004;6:767-76.

43. Westenberg JJ, Doornbos J, Versteegh MI, Bax JJ, van der Geest RJ, de Roos A, Dion RA and Reiber JH. Accurate quantitation of regurgitant volume with MRI in patients selected for mitral valve repair. *European Journal of Cardio-Thoracic Surgery*. 2005;27:462-6; discussion 467.

44. Westenberg JJ, Roes SD, Ajmone Marsan N, Binnendijk NM, Doornbos J, Bax JJ, Reiber JH, de Roos A and van der Geest RJ. Mitral valve and tricuspid valve blood flow: accurate quantification with 3D velocity-encoded MR imaging with retrospective valve tracking. *Radiology*. 2008;249:792-800.

45. Wong J, Chabiniok R, deVecchi A, Dedieu N, Sammut E, Schaeffter T and Razavi R. Age-related changes in intraventricular kinetic energy: a physiological or pathological adaptation? *American Journal of Physiology - Heart & Circulatory Physiology*. 2016;310:H747-55.

46. Zajac J, Eriksson J, Dyverfeldt P, Bolger AF, Ebbers T and Carlhall CJ. Turbulent kinetic energy in normal and myopathic left ventricles. *Journal of Magnetic Resonance Imaging*. 2015;41:1021-9.

47. Mark M, Furiasse N, Lee DC, Ng J, Carr JC and Goldberger JJ. LA velocities and stasis assessed by 4D flow MRI are closely associated with LAA peak velocities by Doppler TEE in patients with atrial fibrillation. *Journal of Cardiovascular Magnetic Resonance Conference: 19th Annual SCMR Scientific Sessions Los Angeles, CA United States Conference Start*. 2016;18.

48. Steding-Ehrenborg K, Arvidsson PM, Rydberg M, Carlsson M and Arheden H. Atrial and ventricular kinetic energy is higher in athletes compared to healthy controls and contributes to improve diastolic filling of the ventricles. *Journal of Cardiovascular Magnetic Resonance Conference: 18th Annual SCMR Scientific Sessions Nice France Conference Start*. 2015;17.

49. Carr M, Ng J, Lee D, Jarvis K, Schnell S, Carr J, Goldberger JJ and Markl M. Atrial fibrillation alters both left and right atrial 3D hemodynamics. *Circulation Conference: American Heart Association's*. 2014;130.

50. Contaldi C, Choudhury L, Allen B, Lee DC, Furiasse N, Puthumana J, Carr J, Markl M and Bonow RO. Left atrial blood flow velocity distribution in hypertrophic cardiomyopathy: Association with left ventricular diastolic function and interstitial fibrosis. *Circulation Conference: American Heart Association's*. 2014;130.

51. Lee DC, Markl M, Fluckiger J, Ng J, Carr JC, Collins JD and Goldberger JJ. Left atrial blood stasis by 4D flow MRI correlates with stroke risk estimation by CHA2DS2-VASc score. *Journal of Cardiovascular Magnetic Resonance Conference: 17th Annual SCMR Scientific Sessions New Orleans, LA United States Conference Start*. 2014;16.

52. Ng J, Markl M, Lee DC, Carr ML, Ng JK, Carr JC and Goldberger JJ. Structural remodeling determinants of reduced left atrial blood flow measured by 4D flow MRI in patients with atrial fibrillation. *Circulation Conference: American Heart Association's*. 2014;130.

53. Fluckiger JU, Goldberger JJ, Lee DC, Ng J, Lee R, Olsen AB, Carr J and Markl M. Quantification of left atrial flow velocity distribution in atrial fibrillation using 4D flow MRI. *Journal of Cardiovascular Magnetic Resonance*. 2013;15:409-410.

54. Goldberger JJ, Fluckiger J, Lee D, Ng J, Olsen A, Carr J and Markl M. Left atrial flow velocity distribution in atrial fibrillation by 4D flow MRI: A new marker for risk of stroke? *Heart Rhythm*. 2013;1):S384.

55. Lee DC, Goldberger JJ, Fluckiger J, Ng J, Carr JC, Collins JD and Markl M. Analysis of left atrial flow velocity distribution by 4D flow MRI in patients with atrial fibrillation. *Circulation Conference: American Heart Association*. 2013;128.

56. Saitoh T, Suwa K, Takehara Y, Sano M, Nobuhara M, Saotome M, Urushida T, Katoh H, Satoh H and Hayashi H. Analyses of intra-left atrial vortex and flow dynamics from pulmonary veins to left ventricle using phase-resolved three-dimensional cine contrast Magnetic Resonance Imaging (4D-FlowMRI). *European Heart Journal*. 2013;34:523-524.

57. Erixon H, Eriksson J, Bolger A, Ebbers T, Karlsson L and Carlhall CJ. 4D flow CMR detects progressive improvement in ventricular function following cardioversion of atrial fibrillation. *Journal of Cardiovascular Magnetic Resonance Conference: 19th Annual SCMR Scientific Sessions Los Angeles, CA United States Conference Start*. 2016;18.

58. Saru RG, Wanambiro K, Hsiao A, Boccalini S, Coenen A, Budde R, Wielopolski P, Vasanawala S, Roos-Hesselink J and Nieman K. Global left ventricular function quantification with CMR 4D Flow. *Journal of Cardiovascular Magnetic Resonance Conference: 19th Annual SCMR Scientific Sessions Los Angeles, CA United States Conference Start*. 2016;18.

59. Stoll V, Hess A, Eriksson J, Rodgers C, Clarke W, Dyverfeldt P, Ebbers T, Myerson S, Carlhall CJ and Neubauer S. 4D flow cardiovascular magnetic resonance parameters represent novel imaging biomarkers in heart failure and correlate with conventional prognostic remodelling parameters and myocardial energetics. *European Journal of Heart Failure*. 2016;18:29-30.

60. Toger J, Kanski M, Arvidsson PM, Carlsson M, Kovacs SJ, Borgquist R, Revstedt J, Soderlind G, Arheden H and Heiberg E. Vortex-ring mixing as a measure of diastolic function of the human heart: Phantom validation and initial observations in healthy volunteers and patients with heart failure. *Journal of Magnetic Resonance Imaging*. 2016;43:1386-1397.

61. Zajac J, Eriksson J, Dyverfeldt P, Alehagen U, Ebbers T, Bolger A and Carlhall CJ. Left ventricular kinetic energy as a marker of mechanical dyssynchrony in failing hearts with LBBB: A 4D flow CMR study. *Journal of Cardiovascular Magnetic Resonance Conference: 19th Annual SCMR Scientific Sessions Los Angeles, CA United States Conference Start*. 2016;18.

62. Hussaini SF, Roldan-Alzate A and Francois CJ. Left and right ventricular kinetic energy using time-resolved versus time-average ventricular volumes. *Journal of Cardiovascular Magnetic Resonance Conference: 18th Annual SCMR Scientific Sessions Nice France Conference Start*. 2015;17.

63. Stoll V, Hess AT, Bissell MM, Eriksson J, Dyverfeldt P, Ebbers T, Myerson SG, Carlhall CJ and Neubauer S. Reproducibility and variability of left ventricular 4D flow in healthy volunteers. *Journal of Cardiovascular Magnetic Resonance*. 2015;17:P7.

64. Chu L, Corona-Villalobos CP, Gulsun MA, Shea S, Markl M, Abraham T, Bluemke D, Kamel IR and Zimmerman SL. Evaluation of left ventricular outflow tract obstruction with 4D phase contrast in patients with hypertrophic cardiomyopathy. *Journal of Cardiovascular Magnetic Resonance Conference: 17th Annual SCMR Scientific Sessions New Orleans, LA United States Conference Start*. 2014;16.

65. El Baz MS, Calkoen E, Westenberg JJ, Lelieveldt BPF, Roest A and Van Der Geest RJ. Three dimensional right ventricular diastolic vortex rings: Characterization and comparison with left ventricular diastolic vortex rings from 4D flow MRI. *Journal of Cardiovascular Magnetic Resonance Conference: 17th Annual SCMR Scientific Sessions New Orleans, LA United States Conference Start*. 2014;16.

66. Zhang D, McGarvey JR, Lee M, Takebayashi S, Aoki C, Dillard C, Contijoch F, Zsido G, Han Q, Han Y, Pilla JJ, Gorman JH, Witschey WR and Gorman RC. Mitral valve stenosis and left ventricular hemodynamic alterations after mitral valve repair. *Journal of Cardiovascular Magnetic Resonance Conference: 17th Annual SCMR Scientific Sessions New Orleans, LA United States Conference Start*. 2014;16.

67. Zajac J, Eriksson J, Dyverfeldt P, Bolger A, Ebbers T and Carlhall CJ. Turbulent kinetic energy from CMR identifies disturbed diastolic flow in myopathic left ventricles. *Journal of Cardiovascular Magnetic Resonance*. 2013;15:88.

68. Eriksson J, Bolger A, Ebbers T and Carlhall CJ. Equal stroke volumes, different costs: Left ventricular 4D flow in normal and failing hearts. *Journal of Cardiovascular Magnetic Resonance Conference: 15th Annual SCMR Scientific Sessions*. 2012;14.

69. Garg P, Hassell ME, Ripley DP, Dobson LE, Swoboda P, Musa TA, Erhayiem B, Haaf P, Greenwood JP, Nijveldt R, Westenberg JJ, Van Der Geest RJ and Plein S. Reliability and reproducibility of trans-valvular flow measurement by 4D flow magnetic resonance imaging in acute myocardial infarct patients: Two centre study. *Journal of Cardiovascular Magnetic Resonance Conference: 19th Annual SCMR Scientific Sessions Los Angeles, CA United States Conference Start*. 2016;18.

70. Lessick J, Gorodisky L and Porat M. Evaluation of mitral regurgitation severity by proximal isovelocity surface area (PISA) method using cardiac magnetic resonance imaging 3D velocity vectors. *European Heart Journal*. 2014;35:121.

71. Nordmeyer S, Riesenkampff E, Messroghli D, Kropf S, Nordmeyer J, Berger F and Kuehne T. Four-dimensional velocity-encoded magnetic resonance imaging improves blood flow quantification in patients with complex accelerated flow. *Journal of Magnetic Resonance Imaging*. 2013;37:208-16.

72. Roldan-Alzate A, Francois C, Wieben O, Chesler NC and Frydrychowicz A. 4D flow-sensitive MR estimation of pulmonary vascular resistance. *Journal of Cardiovascular Magnetic Resonance*. 2013;15:307-308.

73. Fredriksson AG, Svalbring E, Eriksson J, Dyverfeldt P, Alehagen U, Engvall J, Ebbers T and Carlhall CJ. 4D flow MRI can detect subtle right ventricular dysfunction in primary left ventricular disease. *Journal of Magnetic Resonance Imaging*. 2016;43:558-565.

74. Fenster B, Browning J, Stalder AF, Glielmi C, Silveira L, Buckner JK, Kluiber A, Schroeder JD and Hertzberg J. Vorticity for the assessment of right ventricular diastolic dysfunction using 4D flow CMR. *Journal of Cardiovascular Magnetic Resonance*. 2013;15:206-207.

75. Kheyfets VO, Smyser J, Honeyman A, Browning J, Hertzberg JR, Schroeder J, Fenster B and Shandas R. Irregular blood flow patterns in the development of pulmonary hypertension. *Circulation Conference: American Heart Association's*. 2014;130.
